# The Role of Viscoelastic Testing in Assessing Hemostasis: A Challenge to Standard Laboratory Assays?

**DOI:** 10.3390/jcm13123612

**Published:** 2024-06-20

**Authors:** Benjamin Reardon, Leonardo Pasalic, Emmanuel J. Favaloro

**Affiliations:** 1 School of Medicine and Public Health, Joint Medical Program, University of Newcastle, Callaghan, NSW 2145, Australia; benjamin.reardon@health.nsw.gov.au; 2 Haematology Department, Calvary Mater Hospital Newcastle, Waratah, NSW 2298, Australia; 3 Haematology Department, Institute of Clinical Pathology and Medical Research (ICPMR), NSW Health Pathology, Westmead Hospital, Westmead, NSW 2145, Australia; leonardo.pasalic@health.nsw.gov.au; 4 Westmead Clinical School, University of Sydney, Westmead, NSW 2145, Australia; 5 Sydney Centres for Thrombosis and Haemostasis, Westmead Hospital, Westmead, NSW 2145, Australia; 6 School of Dentistry and Medical Sciences, Faculty of Science and Health, Charles Sturt University, Wagga Wagga, NSW 2650, Australia; 7 School of Medical Sciences, Faculty of Medicine and Health, University of Sydney, Westmead Hospital, Westmead, NSW 2145, Australia

**Keywords:** viscoelastic testing, coagulation, hemostasis, transfusion

## Abstract

Viscoelastic testing is increasingly being used in clinical and research settings to assess hemostasis. Indeed, there are potential situations in which viscoelastic testing is reportedly superior to standard routine laboratory testing for hemostasis. We report the current testing platforms and terminology, as well as providing a concise narrative review of the published evidence to guide its use in various clinical settings. Notably, there is increasing evidence of the potential utility of viscoelastic testing for assessment of direct oral anticoagulants, and bleeding associated with chronic liver disease, orthotopic liver transplantation, cardiac surgery, trauma, obstetrics and pediatrics.

## 1. Introduction and Principles of Viscoelastic Testing

**Figure 1:**
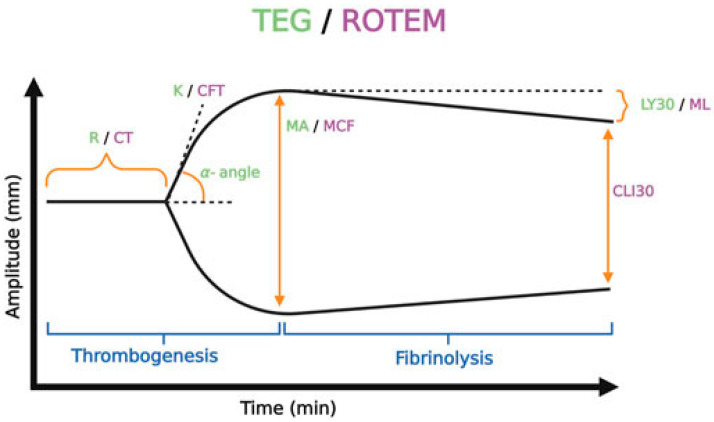
Typical VET waveform (source [22]).

Viscoelastic testing (VET) represents a real-time whole blood assessment of ex-vivo coagulation, reflecting the interaction between plasma, platelets and other blood cells involved in hemostasis [1]. VET includes both Thromboelastography (TEG^®^), Rotational Thromboelastography (ROTEM^®^) and several other more recent testing platforms [2,3,4,5,6]. The basic principle of classical VET involves electromagnetic or other sensors that allow detection of the torque of a pin which sits in a cup of liquid, initially with non-clotted blood, with varying methods which differ according to the manufacturer; notably, in TEG^®^ or ClotPro^®^ compared with ROTEM^®^, whether the pin or the cup oscillates with time. Additionally, the TEG 6S^®^, Sonoclot^®^ and Quantra^®^ devices now utilize resonance-frequency technology and light-emitting diode detection, removing the requirement of a pin and cup mechanism [7,8]. As a clot starts to form, VET allows for assessment of different variables of clot formation but produces different result outputs depending on the chosen instrument. Parameters assessed include time to clot formation (R/CT time), speed of clot formation (alpha angle), clot strength (MA [maximum amplitude] or MCF [maximum clot firmness]) and clot disintegration or lysis (LY30 and LY60, or CLT) [9]. Each of these components act as activity markers of plasma protein hemostasis function, platelet activity, fibrinogen and fibrinolysis, respectively [9,10]. The main parameters assessed by TEG^®^ and ROTEM^®^ systems, and their significance, are listed below and summarized in Table 1. Notably, given the differences in activators used, the results of these devices are not interchangeable. In addition, Figure 1 demonstrates the typical waveform obtained using VET with descriptive measures.

There are many limitations to standard assays of coagulation. Standard prothrombin (PT) and activated partial thromboplastin time (aPTT) are performed using supraphysiological activators to induce clot formation, which may not reflect in vivo hemostasis [11]. A key difference in VET is that it allows for overall assessment of hemostasis rather than testing specific portions of the clotting cascade in isolation [11]. Standard coagulation assays measure time to clot formation measured through a reduction in light transmission (optical systems) or mechanical impedance (mechanical systems), but do not provide information regarding clot strength and stability over time, which VET assays can provide. Plasma-based coagulation assays also fail to assess the contribution of platelets and fibrinolysis, which again can be assessed to some extent by VET assays. Therefore, for standard hemostasis assessment, additional assays to PT and aPTT may be needed to assess all components of hemostasis, including regulatory antithrombic proteins. These additional tests can be laboratory staff labor intensive, time-consuming, have long turn-around times, with testing sometimes only available within centralized laboratories, and finally, overall costs can be high [11,12]. Appropriate hemostatic interventions in bleeding patients are time critical, with delays in the assessment of hemostasis having significant impact on patient care [13]. Despite the fact that standard assays of coagulation are widely available and commonly used, there is very limited published evidence in their role in guiding hemostatic therapy in bleeding patients [14].

VET measures clot formation in real-time using whole blood, and reflecting point of care instruments, often in close proximity to patient care areas with faster turnaround times that standard assays of coagulation [15,16]. Using whole blood has the benefit of not requiring processing time (including centrifugation) prior to analysis, potentially saving critical time in bleeding patients. VET is performed with concurrent whole blood reactions that allow for simultaneous measurement of clotting and fibrinolysis, using various activators (e.g., tissue factor, kaolin) and inhibitors (e.g., abciximab, cytochalasin) to help isolate the various contributions of clotting factors, platelets and fibrinogen. Although the high cost of cartridge-based testing in VET has been an issue for uptake in the past, the introduction of newer platforms and its growing evidence base is increasing its support and reducing overall costs, also noting cost offsets with better more targeted patient management. A notable limitation of VET, as well as standard assays of coagulation, are their inability to assess the contribution of endothelial function in hemostasis [17].

There are multiple different assays used for clot activation or specific component inhibition to identify defects in various aspects of clot formation. Both the TEG^®^ and ROTEM^®^ commonly employ citrated whole blood that is re-calcified to initiate coagulation [18]. Within ROTEM^®^, TEG^®^ or ClotPro^®^ systems there are separate activators or inhibitors including phospholipid and ellagic acid (for use in INTEM or IN-test assays), tissue factor (EXTEM or EX-test assays), kaolin and tissue factor (rapidTEG assay), lyophilised heparinase for heparin neutralization (HEPTEM or HI-test assays), cytochalasin D (FIBTEM assay in ROTEM^®^ only) or glycoprotein IIb/IIIa inhibitor (abcixumab in TEG^®^ only) or combination cytochalasin D and glycoprotein IIb/IIIa inhibition for platelet inhibition (FIB-test in ClotPro^®^); and, aprotinin or tranexamic acid for inhibiting fibrinolysis (APTEM assay in ROTEM^®^) [19,20]. Notably, the TEG 6S^®^ as a more recently available platform enables multiple assays to be simultaneously performed, rather than separate blood aliquots needing to be used for each specific assay. There are also now available additional assays in ClotPro^®^ and other platforms to detect the presence of antifibrinolytic therapies (e.g., TPA-test), the presence of direct oral anticoagulants (DOACs, e.g., RVV-test) and direct thrombin antagonists (e.g., ECA-test) [19].

In recent years, there has been significant increase in research with VET within specific patient subpopulations, leading to increases in the clinical use of VET to direct corrective transfusion-based management in conjunction with standard laboratory tests of coagulation [21]. We present here a concise review of current published data in the use of VET as a means to assess hemostasis or coagulation within clinical groups of interest: direct oral anticoagulants, liver disease, orthotopic liver transplantation, cardiac surgery, trauma, obstetrics and pediatrics.

## 2. Evidence for Use of VET

### 2.1. Direct Oral Anticoagulants

The use of direct oral anticoagulants (DOACs) has significantly increased in recent years, for indications of venous and arterial thromboembolism, prevention of stroke in atrial fibrillation and secondary cardiovascular prevention [23,24,25]. DOACs are taken orally at fixed dosing owing to their stable pharmacokinetics and improved safety profile compared with vitamin K antagonists [26]. Assessment of residual DOAC activity and its impact on hemostasis is essential during acute bleeding or prior to invasive procedures [27]. Current assays of DOAC include chromogenic anti-Xa (with drug-specific calibration curves) and anti-IIa assays as well as less commonly used high-pressure liquid chromatography tandem mass spectrometry (HPLC-MS) [26]. A key issue to many of these assays, particularly in some geographic localities or in smaller centers, is limited availability with centralized processing, despite decision-making being time critical.

A systematic review by Sahil et al. [28] including 53 studies (31 of which assessed rivaroxaban, 22 apixaban, 6 edoxaban, 29 dabigatran) found that plasma concentration of rivaroxaban and dabigatran correlated strongly with VET, but that clotting time (CT) andreaction time (R) within the reference interval did not reliably exclude relevant residual DOAC plasma levels, limiting the clinical utility of VET [28]. Despite this, the use of DOAC-specific cartridges (anti-Xa cartridge or direct thrombin inhibitor channel) have improved the correlation with drug-specific anti-Xa and anti-IIa activity [29]. Oberladstätter et al. (2021) investigated the detection of factor Xa and factor II inhibitors in 203 samples using ecarin clotting time and Russell’s viper venom within the ClotPro^®^ system, respectively, and compared it with plasma-based clotting and chromogenic assays. They found strong linear correlations in these assays (r = 0.9693 for ecarin clotting time and plasma concentration of dabigatran; r = 0.7391 for apixaban, r = 0.8792 for rixaroxaban and r = 0.9251 for edoxaban in Russell’s viper venom and plasma drug levels) [30]. Clinically meaningful cut off drug levels based on the ClotPro^®^ system demonstrated varying but overall high levels of both sensitivity and specificity across all drugs tested. DOAC blood concentrations have also been correlated with TEG6S^®^ using the direct thrombin inhibitor and anti-factor Xa channels with strong correlations in health male volunteers (for dabigatran levels *r* = 0.94, *p* < 0.0001, and for rivaroxaban and apixaban *r* = 0.93 and *r* = 0.83, respectively; *p* < 0.0001 for both) [29]. Similar results have also been demonstrated by Bliden et al. 2017 [31].

Direct reversal agents Idarucizumab and andexanet alfa have been developed as inhibitors active against dabigatran and anti-Xa based DOACs, respectively. Takeshita et al. [32] investigated the reversal of dabigatran in two VET assays as well as with aPTT and PT, and this resulted in reversal of previously abnormal VET parameters INTEM (ellagic acid) and EXTEM (tissue factor) with idarucizumab at all dabigatran concentrations (0, 200, 500, 1000, 1500, 2000, 3000, and 5000 ng/mL) [32]. Oberladstätter et al. (2021) have also assessed the ex vivo effect spiking in 37 patients and 10 health volunteers and found that the Russell viper venom clot time (RVV-CT) using ClotPro^®^ decreased but did not normalize following administration of Andexanet alfa [33]. Interestingly Andexanet alfa spiking in non-anticoagulated blood resulted in prolonged RVV-CT (*p* = 0.001). The authors suggested that chemical similarities between andexanet alfa and endogenous factor Xa may lead to competitive antagonism resulting in only partial inhibition of the coagulation process rather than completely normalizing it, as well as inducing prolongation in non-anticoagulated blood. In vitro spiking of dabigatran-containing blood with Idarucizumab decreased the measured dabigatran level as well as the ecarin-test clotting time using ClotPro^®^ (all, *p* < 0.001) [33].

### 2.2. Liver Disease

Chronic liver disease and cirrhosis often leads to abnormal ex vivo coagulation tests, despite rebalanced hemostasis, but also may sometimes present clinically as either pro-thrombotic or pro-hemorrhagic phenotypes [34]. VET has been increasingly studied in patients with chronic liver disease as an alternative to standard coagulation testing with varying success. Standard coagulation assays fail to identify liver disease subgroups and often shows abnormalities in many phases of hemostasis [35]. The common abnormalities seen in liver disease are prolonged PTs through reduced production of vitamin K dependent clotting factors, quantitative thrombocytopenia by mechanisms of platelet spleen sequestration, low thrombopoietin levels and immune destruction, and hypo/dysfibrinogenemia [1,36]. Standard tests of PT/international normalized ratio (INR) and fibrinogen are not recommended for assessment prior to high-risk bleeding procedures in patients with cirrhosis by the International Society on Thrombosis and Haemostasis [37], European association for the Study of the Liver [38] and the American Gastroenterological Association [39]. Compensatory increases in factor VIII activity and von Willebrand factor (VWF), with reduced ADAMTS13 are seen in chronic liver disease and may contribute to increased platelet adherence despite quantitative platelet reduction [33,40]. Some guidelines also suggest that platelet count is not required prior to high-risk bleeding procedures in patients with cirrhosis [37,38,41].

The use of VET in cirrhosis has produced varying results. Hugenholtz (2017) found that VET did not predict future bleeding or thrombotic events compared with standard coagulation tests in patients with cirrhosis [42]. However, Chau et al. (1998) previously demonstrated that serial VET testing in the first seven days following sclerotherapy and banding for patients with cirrhosis who presented with gastric variceal bleeding reliably identified abnormalities the day prior to rebleeding events that standard coagulation assays did not [43]. Lisman (2020) also highlights the propensity of VET to underestimate patients’ coagulation capacity in chronic liver disease and cirrhosis given VETs intrinsic inability to assess endothelial cell function of thrombomodulin and insensitivity to protein C activity, which are key compensatory mechanisms to rebalance hemostasis in this population [13]. The lack of flow movement in VET may underestimate VWF activity as a platelet-adhesive protein, which is increased in chronic liver disease. As a result, Lisman suggests that transfusion triggers should be increased in patients with chronic liver disease [17].

A randomized controlled trial by De Pietri et al. (2016) randomly assigned 60 patients with cirrhosis to a VET-based transfusion strategy compared with standard coagulation-based approach prior to planned invasive procedures [44]. They found that patients within the standard of care arm had higher transfusion requirements for fresh frozen plasma (FFP, 53.3% of patients compared with 0%, *p* < 0.00001) and platelets (33.3% compared with 6.7%, *p* = 0.021) compared with VET-based patients, with no increase in bleeding complications post procedure. A subsequent meta-analysis by Tangcheewinsirikul et al. (2022) analyzed acute bleeding events in patients with chronic liver disease from 7 randomized controlled trials (*n* = 421), and found that the VET-guided transfusion algorithm yielded a reduction in the transfused total volume of FFP (RR 0.52; 95% CI 0.35–0.77) and platelet transfusion (RR 0.34; 95% CI 0.16–0.73) compared with standard of care [45]. Patients with VET-guided transfusion strategy also had a reduced risk of transfusion-related adverse effects with no differences seen in post procedural bleeding rates (RR 1.05; 95% 0.94–1.17).

### 2.3. Orthotopic Liver Transplantation

Assessment of coagulation and appropriate transfusion management in patients undergoing orthotopic liver transplantation is challenging, with significant risks associated with large volumes of transfused blood products [46]. There is questionable utility in conventional coagulation testing in this population, including the use of PT and aPTT to assess bleeding risk [47].

A non-randomized prospective study of 60 patients by Roullet et al. (2015) of VET-based transfusion algorithm compared with standard of care of patients undergoing orthotopic liver transplantation found no differences in volumes of transfused red cells or FFP, with a small non-significant increase in the median amount of fibrinogen given in the VET-based group (6.0 g vs. 4.5 g, *p* = 0.50) with no difference in post operative bleeding rates [48]. There were fewer platelets transfused in the VET-based group (*n* = 13 vs. 15). There were a number of limitations in this study, including low patient numbers, lack of VET use in the ICU setting in the immediate post operative period, and lack of VET-specific plasma protein assessment, where plasma was given at discretion of the clinician. Wang et al. (2010) performed a randomized trial comparing VET (TEG^®^) to standard of care coagulation test guided hemostatic management in 28 adult patients undergoing orthotopic liver transplantation and found that the VET based approach was associated with lower usage of FFP (mean 12.8 units +/− 7 compared with 21.5 units +/− 12.7; *p* < 0.05) and a trend towards less blood loss without any difference in total fluid administration and 3-year overall survival [49]. In 2019, Bonnet et al. randomized 81 adult patients undergoing orthotopic liver transplantation to also compare VET (ROTEM) to standard of care coagulation test guided hemostatic management and found that the median number of red cell units, FFP and platelet transfusion did not differ between groups, with greater rates of fibrinogen administration in the VET-directed group [50]. FFP and tranexamic acid were administered less frequently in the VET (ROTEM) group (respectively 15% vs. 46.3%, *p* = 0.002; and 27.5% vs. 58.5%, *p* = −0.005). A later non-randomized study by Nascimento (2020) found that 82 patients undergoing orthotopic liver transplantation with VET directed transfusion management had lower FFP usage, with equivalent rates of red cell transfusion, but higher rates of fibrinogen and prothrombin complex concentrate administration compared with standard coagulation testing-based transfusion management [51].

Overall, VET-based assessment of coagulation may lead to reductions in red cell and FFP administration in patients undergoing orthotopic liver transplantation, with perhaps a potential increase in fibrinogen and platelet transfusion usage. VET based transfusion strategies have now been introduced into multiple guidelines for patient’s undergoing orthotopic liver transplantation [52,53,54]. The complexities of assessment with VET and chronic liver disease still stand for this population peri-procedurally regarding potential underestimation of overall hemostatic capacity [17].

### 2.4. Cardiothoracic Surgery

The comparison of VET with standard assays of coagulation has most commonly been assessed following cardiothoracic surgery, where hemostatic abnormalities are common. Bleeding is a common complication following cardiothoracic surgery, with reports of bleeding complications in as high as 20% of cases. There are many factors that can contribute to bleeding in this population including significant hemodilution, platelet dysfunction, use of large doses of intraoperative anticoagulation, the use of cardiopulmonary bypass itself and post operative fibrinolysis [55].

In 2016, a Cochrane Database systematic review of 17 randomized controlled trials, with a total number of patients 1493, demonstrated that VET-guided transfusion management reduced red blood cell, FFP and platelet transfusion [56]. In a large randomized controlled trial with 3847 patients from 12 different hospitals, the VET based transfusion strategy compared with standard coagulation assay-based care post cardiac surgery reduced the rate of major bleeding (RR 0.83, CI 95% 0.72–0.94, *p* = 0.004; number needed to treat, 22.6) with a 26% reduction in the rate of patients requiring reoperation, despite a greater percentage of surgeries being considered ‘complex cardiac surgeries’, with a reduction in the amount of red blood cell and platelet transfusions [57]. Findings of reduced red cell and FFP use with VET-guided transfusion strategy following cardiac and thoracic surgery was also found by Li et al. (2019) [58] and Meco et al. (2020) [59], as well as by Deppe et al. (2016) [60] in a large meta-analysis of 8332 patients whereby VET-guided transfusion management significantly reduced the odds of patients receiving allogenetic blood products (OR 0.63, 95% CI 0.56–0.71, *p* < 0.00001) and the re-exploration rate due to post-operative bleeding (OR 0.56, 95% CI 0.61–0.98). Kuiper et al. (2019) found that the use of a VET-based transfusion algorithm at their single center was associated with a reduced average blood loss (325 mL less with VET), a 30% relative reduction in the number of patients given red cell transfusion, a 47% relative reduction in patient transfusion overall, with 4 days fewer in hospital, but with no differences in average hemoglobin concentration, cryoprecipitate use or length of ICU stay compared with patients treated based on a conventional coagulation assay algorithm [61]. A meta-analysis of 21 randomized trials from Santos et al. (2020) demonstrated that VET-guided transfusion and assessment of coagulation post cardiac surgery was associated with reduction in mortality, risk of acute kidney injury and transfusion reaction compared with standard of care, including use of standard coagulation assays [62].

Viscoelastic testing has also been used in thoraco-abdominal surgery. In a small case series of 18 patients undergoing thoraco-abdominal aortic aneurysm repair, ROTEM-guided use of fibrinogen concentrate reduced the total transfusion units in 6 patients (2.5 vs. 16.4 total units) with reduced 24-h drainage volume (449 vs. 1092 mL), compared with 12 retrospective controls [63]. The use of VET in the context of aortic dissection is less clear. In pre-operative assessment of patients with acute Stanford type A aortic dissection, there has been no described role of VET in predicting post operative hemorrhage and transfusion requirements [64,65]. However, the perioperative use of VET in those who require cardiopulmonary bypass (CPB) has been less well validated in the context of aortic surgery, but recommended by thoracic surgery guidelines [66,67]. The use of VET in monitoring patients following CBP bypass has been validated in other cardiac surgeries [68] but is recommended as a key component in management of aortic dissection surgery [65].

Antiplatelet agents and identification of platelet dysfunction is a common issue in patients undergoing vascular and cardiac surgeries. The adaption of TEG platelet mapping (TEG PM^®^) utilizing heparinized blood treated with ADP and combination reptilase and factor XIIIa, and comparison with non-heparinized blood of the same patient, can be used to measure clot strength due to remaining platelet function [69]. TEG PM has been shown to predict excessive postoperative bleeding in patients who are on anti-platelet therapy following cardiac surgery [70], and reliably predict platelet dysfunction in patients taking antiplatelet therapy prior to non-cardiac surgery [71]. As a result, the Society of Thoracic Surgeons [66] and the European Association of CardioThoracic Anaesthetists [67] recommend platelet function testing in patients taking antiplatelet agents prior to cardiothoracic surgery to guide the timing of surgery.

### 2.5. Trauma

Presentations of trauma are inherently associated with bleeding, both from blunt and penetrating injuries. Secondary massive hemorrhage can lead to hypovolemic shock with sympatho-adrenal activation, endothelial activation and an eventual hypocoagulable and hyperfibrinolytic state, which defines trauma-induced coagulopathy [22].

In a large multi-center randomized controlled trial (iTACTIC), 201 patients presenting with traumatic injuries were allocated to VET-guided transfusion algorithm compared with 195 patients allocated to conventional coagulation testing-guided therapy [72]. The authors found that there were no differences in mortality or massive transfusion at 24 h and no difference in overall mortality at 28 days in VET-assessed patients. In total, 75% of patients had no abnormalities in coagulation at baseline. However, in patients who did present with coagulopathy, there was a trend towards improved outcome with a non-statistically significant *p* value of 0.07. In a pre-specified subgroup of 74 patients with traumatic brain injury, 64% of were alive and free of massive transfusion at 24 h compared with 46% in the control arm (OR 2.12, 95% CI 0.84, 5.34). Interestingly, the incidence of thrombotic complications was lower in VET-managed patients (9% vs. 14%, non-significant).

In a separate single center study of 111 trauma patients who required massive transfusion protocol activation [73], the VET-guided transfusion algorithm was associated with improved overall survival (11 compared with 20 deaths with standard coagulation tests, log-rank *p* = 0.032) with majority of deaths occurring within 6 h from arrival, but with no differences in red cell transfusion use in the VET-directed group compared with standard of care. Interestingly, Goodman et al. (2015) found that there was a high correlation between point of care INR testing and VET in patients presenting with trauma at a single center but they did not assess transfusion practices between the two groups, nor the means of assessment of platelets or fibrinogen, or their associated activity in this population [74]. They concluded that VET was costly and therefore not needed, despite evidence that INR does not necessarily correlate with coagulopathy in trauma patients and that it is a poor management target in this patient group [74,75,76].

### 2.6. Obstetrics

Altered hemostasis in pregnancy may lead to thrombosis or bleeding in the peripartum and postpartum periods. The generalized hypercoagulable state of pregnancy and various pregnancy-associated disease states including pre-eclampsia, gestational diabetes, hemolysis and elevated liver function enzymes with low platelets (HELLP) can be reliably detected by VET [73,77]. VET-associated changes have also been reported following recurrent pregnancy loss [78] as well as fibrinolysis associated with antiphospholipid syndrome [79].

VET parameters have been shown to reliably detect hypofibrinogenemia and thrombocytopenia in postpartum patients [80] as well as the hypercoagulable state peripartum and postpartum [81]. In a prospective cohort study by McNamara et al. (2019), VET-directed transfusion was associated with significantly less circulatory overload in 255 women with peripartum bleeding [82]. VET-directed transfusion prescription has also been shown to have reduced red blood cell, FFP and platelet transfusion as well as a reduced length of stay and ICU admission time in severe post-partum hemorrhage (>1500 mL blood loss) in a retrospective cohort study of 86 women [83]. Beyond peripartum bleeding, the use of VET has been shown to demonstrate adequate platelet function to proceed with neuraxial anesthesia when quantitative platelets are lower than 56,000/mm^3^ [77,84,85].

The differences between ClotPro^®^ and ROTEM^®^ has recently been studied by Gruneberg et al. (2024) in 217 parturient women, whereby differences were seen in extrinsic and intrinsic pathways as well as fibrinogen assays, highlighting the need for device-specific transfusion algorithms given these differences [86].

### 2.7. Pediatrics

The majority of published papers of VET in pediatric populations are retrospective analyses, which focus on correlation to standard coagulation tests or focus on prediction of bleeding, particularly following cardiac surgery [8]. Reference ranges for pediatric age ranges have been reported for ROTEM^®^ [87,88,89], TEG^®^ [90,91,92,93,94] and Sonoclot systems [95]. Haas and Faraoni (2020) suggest that as the maturation of hemostasis occurs within the 1st year of life, adult reference ranges can be applied for pediatric patients over the age of 1 year [8]. The blood volumes required for analysis of VET vary with different devices but are overall less volume compared to standard coagulation testing; this requires consideration given that regular blood testing may further challenge the physiologic reserve of neonates in addition to ongoing blood loss [8].

VET-based transfusion strategies have been reported following pediatric cardiac surgery. In one two-phase randomized trial, Nakayama et al., 2014 found that the ROTEM^®^-guided transfusion strategy reduced red cell requirements, post operative bleeding and length of ICU stay, compared with conventional coagulation assays, in 178 pediatric patients who underwent cardiac bypass surgery [96]. In a retrospective study by Kim et al., abnormal ROTEM^®^ parameters of FIBTEM A10, EXTEM, alpha angle and ML were independent risk factors for excessive bleeding and were appropriate to guide transfusion therapy [97]. The implementation of a transfusion algorithm in pediatric patients undergoing cardiac surgery has enabled rapid detection of coagulopathy in the presence of excessive bleeding [8].

Following pediatric craniofacial surgery, Haas et al. (2014) found that the mean total cost of bleeding management was reduced when incorporating ROTEM^®^ guided treatment using coagulation factor concentrates with a reduction in 64% of allogeneic blood products used [98].

VET testing in pediatric patients presenting with trauma has been identified as an area of need with a paucity of high-level prospective evidence at present [99]. Early identification of hypofibrinogenemia in trauma with VET has also been identified as an area of need within a pediatric population, particularly when considering off the shelf products [100]. In a retrospective cohort study by Deng et al. (2018), ROTEM-guided treatment in 332 pediatric patients presenting with trauma found that VET-based transfusion strategies had significantly less plasma transfusion in the first 24 h (mean 147 mL vs. 175 mL, *p* = 0.02) and shorter length of stay (11.2 vs. 12.1 days, *p* = 0.02) [101].

VET testing in pediatric liver transplantation has also been evaluated. In a retrospective cohort study, Sujka et al. (2018) found that there was a significant reduction in FFP use (17 mL compared with 111 mL, *p* = <0.01) for pediatric patients who underwent a TEG-based transfusion strategy compared with conventional coagulation assays in 38 patients receiving orthotopic liver transplantation [102].

## 3. Challenges and Future Directions

Clinical use of VET is growing. Adequate understanding and interpretation of VET is critical for measuring its impact, with lack of experience and adherence to complicated VET-based transfusion protocols being a significant limitation to its use. Further analysis is needed to assess the utility of implementing VET-based transfusion algorithms on transfusion requirements in different pediatric populations including neonatal surgeries. VET use in specific bleeding disorders has not been established, particularly regarding von Willebrand disease (VWD). Further research is required to better assess detection of DOAC using VET and their specific inhibitors in the setting of acute bleeding. VET cartridges to assess the effect of direct oral anticoagulants require further analysis.

## Figures and Tables

**Table 1:** Variables assessed by TEG^®^ and ROTEM^®^ systems.

Variable	Significance	TEG^®^	ROTEM^®^
Time [minutes] from start of 2 mm above baseline	Clotting factor deficiency if prolonged	Reaction Time [R]	Clotting time [CT]
Time [minutes] from 2 mm above baseline to 20 mm above baseline	Hypofibrinogemia if prolonged	Kinetics Time [K]	Clot formation time [CFT]
Alpha [α] angle [°]	Measures the slope between clot initiation and formation, representing thrombin propagation	Angle between end of R time and slope of curve	Angle between end of CT to slope of the curve
Maximum strength [mm] and peak amplitude of waveform	Low fibrinogen, low platelets or platelet dysfunction if reduced	Maximal Amplitude [MA]	Maximal clot firmness [MCF]
Time [minutes] to Maximum Clot Firmness	Hypofibrinogemia if prolonged	-	MCF-t
Clot Lysis	Detection of hyperfibrinolysis	CLT [time taken for amplitude to decrease by 2 mm from MA]	LY30, LY45, LY60 [percentage drop in amplitude of MCF at 30, 45 and 60 min]
Clot elasticity [dyn/cm^2^]	Clot ‘firmness’	G	MCE
Maximum Lysis	Detection of hyperfibrinolysis	-	ML

## Data Availability

No new data were created or analyzed in this study. Data sharing is not applicable to this article.
